# Effect of Spatial Disorientation in a Virtual Environment on Gait and Vital Features in Patients with Dementia: Pilot Single-Blind Randomized Control Trial

**DOI:** 10.2196/18455

**Published:** 2020-10-08

**Authors:** Chimezie O Amaefule, Stefan Lüdtke, Thomas Kirste, Stefan J Teipel

**Affiliations:** 1 German Center for Neurodegenerative Diseases (DZNE) Rostock Germany; 2 Institute of Visual & Analytic Computing University of Rostock Rostock Germany; 3 Department of Psychosomatic and Psychotherapeutic Medicine University Medicine Rostock Rostock Germany

**Keywords:** spatial disorientation, activity recognition, wayfinding, wearable sensors, dementia, virtual reality, older adults

## Abstract

**Background:**

Orientation deficits are among the most devastating consequences of early dementia. Digital navigation devices could overcome these deficits if adaptable to the user’s needs (ie, provide situation-aware, proactive navigation assistance). To fulfill this task, systems need to automatically detect spatial disorientation from sensors in real time. Ideally, this would require field studies consisting of real-world navigation. However, such field studies can be challenging and are not guaranteed to cover sufficient instances of disorientation due to the large variability of real-world settings and a lack of control over the environment.

**Objective:**

Extending a foregoing field study, we aim to evaluate the feasibility of using a sophisticated virtual reality (VR) setup, which allows a more controlled observation of disorientation states and accompanying behavioral and physiological parameters in cognitively healthy older people and people with dementia.

**Methods:**

In this feasibility study, we described the experimental design and pilot outcomes of an ongoing study aimed at investigating the effect of disorientation on gait and selected physiological features in a virtual laboratory. We transferred a real-world navigation task to a treadmill-based virtual system for gait analysis. Disorientation was induced by deliberately manipulating landmarks in the VR projection. Associated responses in motion behavior and physiological parameters were recorded by sensors. Primary outcomes were variations in motion and physiological parameters, frequency of disorientation, and questionnaire-derived usability estimates (immersion and perceived control of the gait system) for our population of interest. At this time, the included participants were 9 cognitively healthy older participants [5/9 women, 4/9 men; mean age 70 years, SD 4.40; Mini–Mental State Examination (MMSE) mean 29, SD 0.70) and 4 participants with dementia (2/4 women, 2/4 men; mean age 78 years, SD 2.30 years; MMSE mean 20.50, SD 7.54). Recruitment is ongoing, with the aim of including 30 cognitively healthy older participants and 20 participants with dementia.

**Results:**

All 13 participants completed the experiment. Patients’ route was adapted by shortening it relative to the original route. Average instances of disorientation were 21.40, 36.50, and 37.50 for the cognitively healthy older control, cognitively healthy older experimental participants, and participants with dementia, respectively. Questionnaire outcomes indicated that participants experienced adequate usability and immersion; 4.30 for presence, 3.73 for involvement, and 3.85 for realism of 7 possible points, indicating a good overall ability to cope with the experiment. Variations were also observed in motion and physiological parameters during instances of disorientation.

**Conclusions:**

This study presents the first feasibility outcomes of a study investigating the viability of using a sophisticated VR setup, based on an earlier real-world navigation study, to study spatial disorientation among cognitively healthy older people and people with dementia. Preliminary outcomes give confidence to the notion that our setup can be used to assess motion and physiological markers of disorientation, even in people with cognitive decline.

**Trial Registration:**

ClinicalTrials.gov; https://clinicaltrials.gov/ct2/show/NCT04134806

## Introduction

### Background

Challenges in wayfinding and orientation are early symptoms of people with mild cognitive impairment (MCI) or dementia. These deficits decrease mobility [1,2] and social interaction [3,4] of the affected people, which in turn may lead to further cognitive decline. Assistive technology devices (ATDs) can help reduce the burden of spatial disorientation by providing interventions (eg, by giving cues to the patients). For patients with cognitive impairment, an ideal ATD should fulfill 2 requirements: It should be situation-adaptive (ie, the device adapts to the situation and context, including the environment, user goal, and intention), and it should be subsidiary (ie, the device delivers assistance only in case of need, for example, if the user is disoriented). These requirements ensure that the device does not replace but rather leverages existing cognitive capabilities [5]. Technically, this means that the ATD needs to be able to detect instances of disorientation in real time from the available sensor data, like accelerometric, electrodermal (EDA), or electrocardiographic (ECG) data.

In a previous field study—Situation-Aware Navigation Assistance for Dementia Patients using Causal Behavior Models (SiNDeM) [6]—concerned with wayfinding behavior in MCI and patients with dementia through an urban environment, a machine learning classifier for disorientation based solely on accelerometric data led to a cross-validated area under the receiver operating characteristics curve (AUC) value of 0.75. The outcome of this study suggested that instantaneous detection of disorientation may, in principle, be possible; however, the accuracy was not sufficient to serve as a basis for individual support. Hence, additional signals, such as heart rate variability, may be needed to increase detection accuracy. However, performing such a study in a real-world environment requires a large effort in staff and resources. One of the limitations highlighted in the previous study [6] was that the recorded number of disorientation instances per subject was low, which can be problematic when training machine learning classifiers. This is due to the fact that, firstly, the experimenter could not influence whether (and when) subjects became disoriented. Secondly, the high level of inconsistency in the real-world environment did not enable the controlled observation of disorientation states that are not induced by experimental manipulation (eg, changing of landmarks) but rather by cognitive deficits, as in the case of the patients. Thus, a large effort is required to obtain only a small amount of data related to disorientation instances (which is the data that is most relevant in this context). A more robust approach to modeling real-time disorientation might rely on both a controlled environment as well as other more informative parameters in addition to motion. Gait features have recently been vastly explored as motion markers of disease progression [7-9] and fall detection [10], and could also be informative in identifying behavioral variations predictive of disorientation. Additionally, physiological parameters, including skin conductance response and heart rate variations, have also been previously shown to be influenced by the occurrence of spatial disorientation [11,12].

As an alternative to real-world studies, navigation tasks can be posed in a virtual-reality (VR) environment [13,14]. However, most experimental setups do not integrate a physical component—participants sit in front of a computer screen, and thus the physical manifestation of disorientation cannot be assessed. More generally, the need for physical locomotion might influence navigation behavior, as participants are in a dual-task situation where they have to simultaneously walk and navigate through the environment. Such dual-task conditions require cognitive resources such as attentional flexibility, which are depleted as disease progresses [15-18]. Therefore, a pertinent question would be, how can the navigation task be transferred to a safe and controllable VR environment while the participants still have to walk actively (like they would do in the real world), to allow for the investigation of the relationship between disorientation and physical motion? To this extent, we propose a VR-based experimental setup that allows realistic physical movement.

Specifically, we employed the GRAIL (Gait Real-time Analysis Interactive Lab; Motekforce Link) system, providing the opportunity to navigate on a treadmill through a virtual environment. Using such a virtual environment has several advantages: The setup is safe for the subjects, reproducible, and the experimental effort per subject is lower compared to a real-world study. Furthermore, the experimenters have full control over the environment; for example, the environment can be manipulated to induce disorientation to record a larger amount of disorientation instances. Also, disorientation states resulting from cognitive deficits can be properly observed.

### Objectives

This study aims to evaluate the feasibility of using a complex virtual reality setup, which allows a more controlled observation of disorientation states, to study spatial disorientation among older cognitively healthy people and people with dementia, thereby validating the findings of the real-world SiNDeM study. This environment gives access to a broader set of sensor domains compared with the real-world setting, including gait, skin conductance, and heart rate variability in addition to accelerometry; it also allows for the examination of a larger sample size within a more controlled environment to build a more accurate disorientation detection and intervention model.

### Research Questions

The study is based on the following core research question: Is our setup feasible for investigating states of disorientation during active navigation among cognitively healthy older participants and people with dementia? This is motivated by the reported overall effectiveness of VR in assessing spatial navigation [19], spatial navigation memory for predementia screening [20], and improving cognitive functioning among individuals with neurocognitive disorders [21,22]. Evaluation of the feasibility of our setup is further guided by the following research questions: (1) Is the virtual environment adequately immersive? (2) Are we able to reliably induce disorientation through the manipulation of the virtual environment? (3) Do the participants feel comfortable with the walking pattern change due to our navigation mechanism? (4) Are we able to adequately measure motion and physiological parameters?

## Methods

### Study Design and Setting

**Figure 1:**
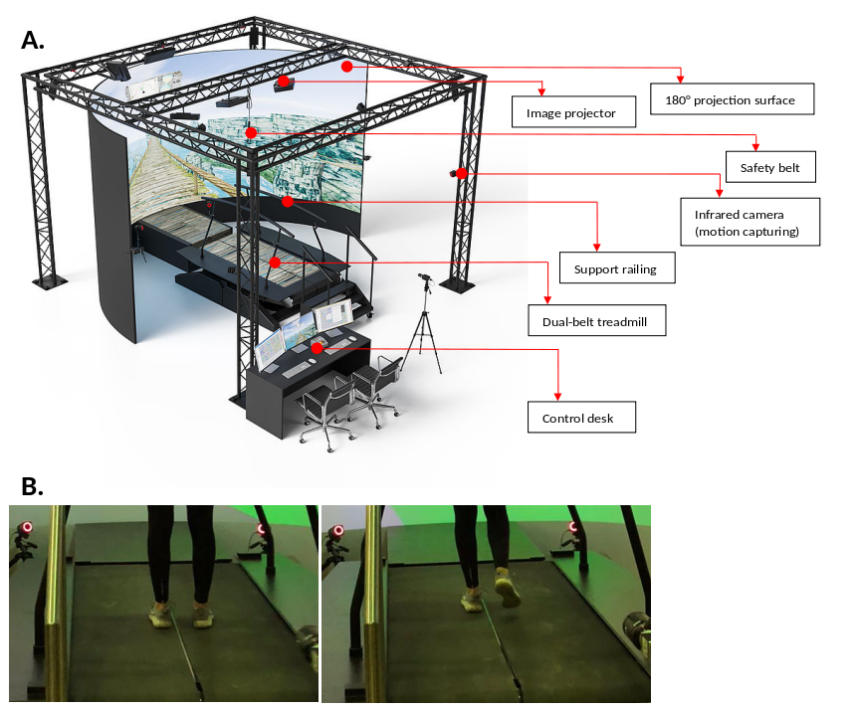
(A) The GRAIL system, consisting of a dual-belt treadmill, 180° projection surface, and optical motion capturing system (figure sourced from Motek Medical); (B) plantar figures.

This is a single-blind randomized experiment currently taking place in Rostock, Germany. The experiments are carried out in the Gait Real-time Analysis Interactive Lab (GRAIL; Motekforce Link; Figure 1), which is a Class I medical system (according to the Medical Device Directive 93/42/EEC) and is specially designed to ensure the safety of participants for clinical gait analysis and training. This is achieved through a safety belt and side railings, which serve to support the participant and prevent falls. The GRAIL further consists of a treadmill, a large 180° projection screen, and an optical motion capturing system (Vicon Motion Systems Ltd). A virtual environment that models a city center is shown onscreen, and subjects can navigate through the environment by walking on the treadmill. Gait kinematics and kinetics, as well as spatio-temporal gait parameters, can be derived from the motion capturing data [23]. The system has been used, for example, for rehabilitation exercise [24] or for gait analysis in different settings [25-27].

**Figure 2:**
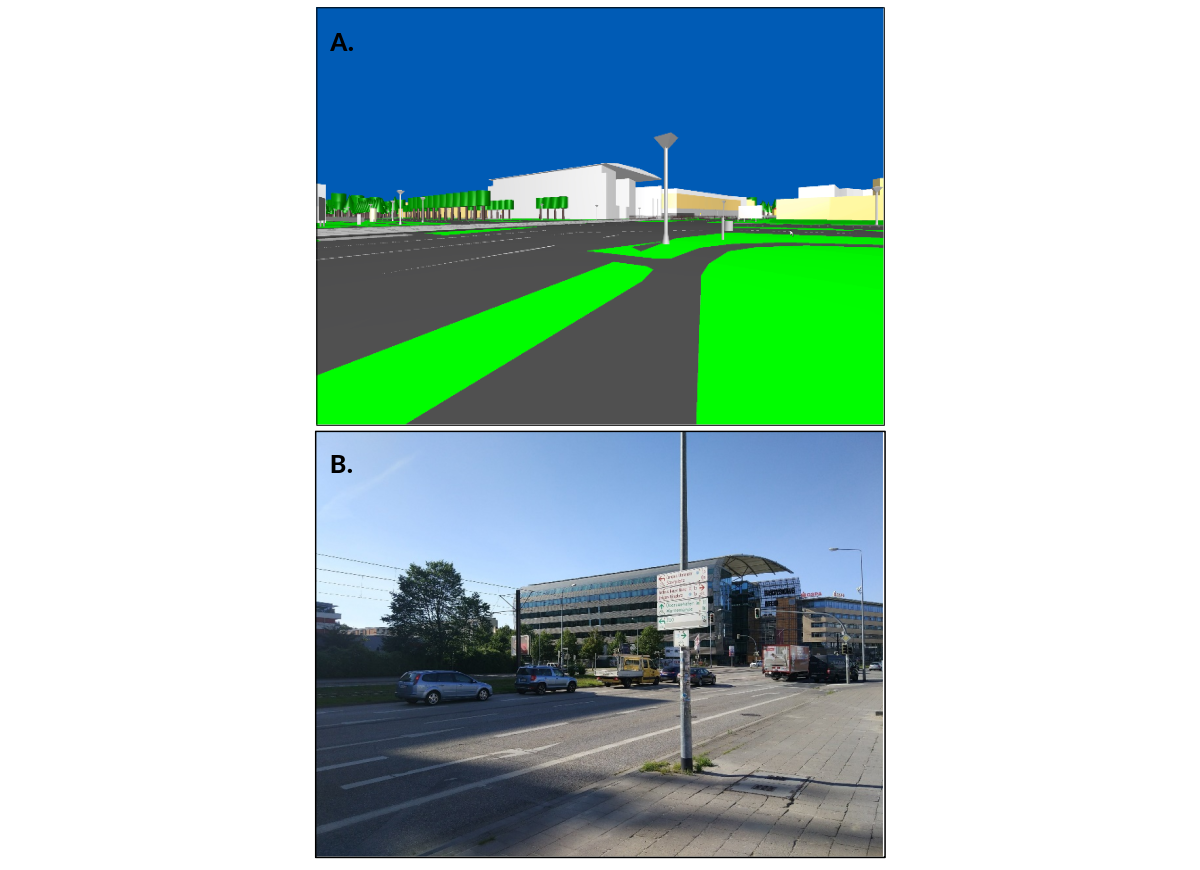
(A) The OpenStreetMap (OSM)–generated virtual environment containing some notable landmarks from (B) the real world.

The 3D virtual environment used in this study was generated from OpenStreetMap (OSM) data of the Rostock city center, using the OSM2World tool [28]. This data includes building heights and rudimentary 3D models of landmark buildings (Figure 2). The resulting VR environment is a low-detail replication of the real city, but does not contain moving objects like cars or pedestrians. The treadmill speed is feedback-controlled, which allows participants to walk with their preferred walking speed. This is achieved by adapting the belt speed according to the subject position (measured by motion capturing) in relationship to the center of the belt [29]. The movement speed through the VR is synchronized with the belt speed (ie, the current speed of the participant). An important constraint of the GRAIL system is that a change in walking direction (eg, turning left or right) is not supported by the treadmill; it does not rotate. Thus, it was necessary to provide an alternative means for voluntary direction change: participants can choose their walking direction by walking on either side of the treadmill. Walking on the left side of the treadmill will result in a left turn in the VR environment, and vice versa. We are aware that this is not identical to naturalistic walking: subjects still walk a straight line on the treadmill, whereas the movement in the VR describes a curve. Therefore, as part of the current study reports, we observed the movement behavior of participants and asked them about their experiences.

### Recruitment and Eligibility

Participants were recruited in 3 groups: (1) mobile, physically and cognitively healthy, younger (18-40 years of age) adults; (2) mobile, physically healthy older [60-85 years of age; Mini–Mental State Examination (MMSE) scores≥28] adults, including cognitively healthy older adults without memory complaints and cognitively healthy older adults with subjective cognitive decline (SCD) in the absence of any clinical evidence of cognitive impairment; and (3) physically healthy older adults with diagnosed MCI or mild dementia due to Alzheimer disease (60-85 years of age; MMSE 15-27). People with dementia and cognitively healthy older adults are recruited from the memory clinic of the University Medicine, Rostock, while the healthy young adults are recruited from within the University of Rostock student community. Exclusion criteria for all groups include other neurological conditions besides dementia, an inability to understand task instructions and questionnaire items, and deaf-mutism and blindness.

As the focus of this feasibility study was on cognitively healthy older adults and people with dementia, 9 cognitively healthy older participants (5/9 women, 4/9 men; 6/9 with SCD; mean age 70 years, SD 4.40; MMSE mean 29, SD 0.70) and 4 people with dementia (2/4 women, 2/4 men; mean age 78 years, SD 2.30; MMSE mean 20.50, SD 7.54) have been included so far. Of the 9 cognitively healthy older participants, 4 participants (2/4 women, 2/4 men) were randomly assigned to the experimental group. Informed consent was given by all participants.

### Study Procedure and Data Collection

**Figure 3:**
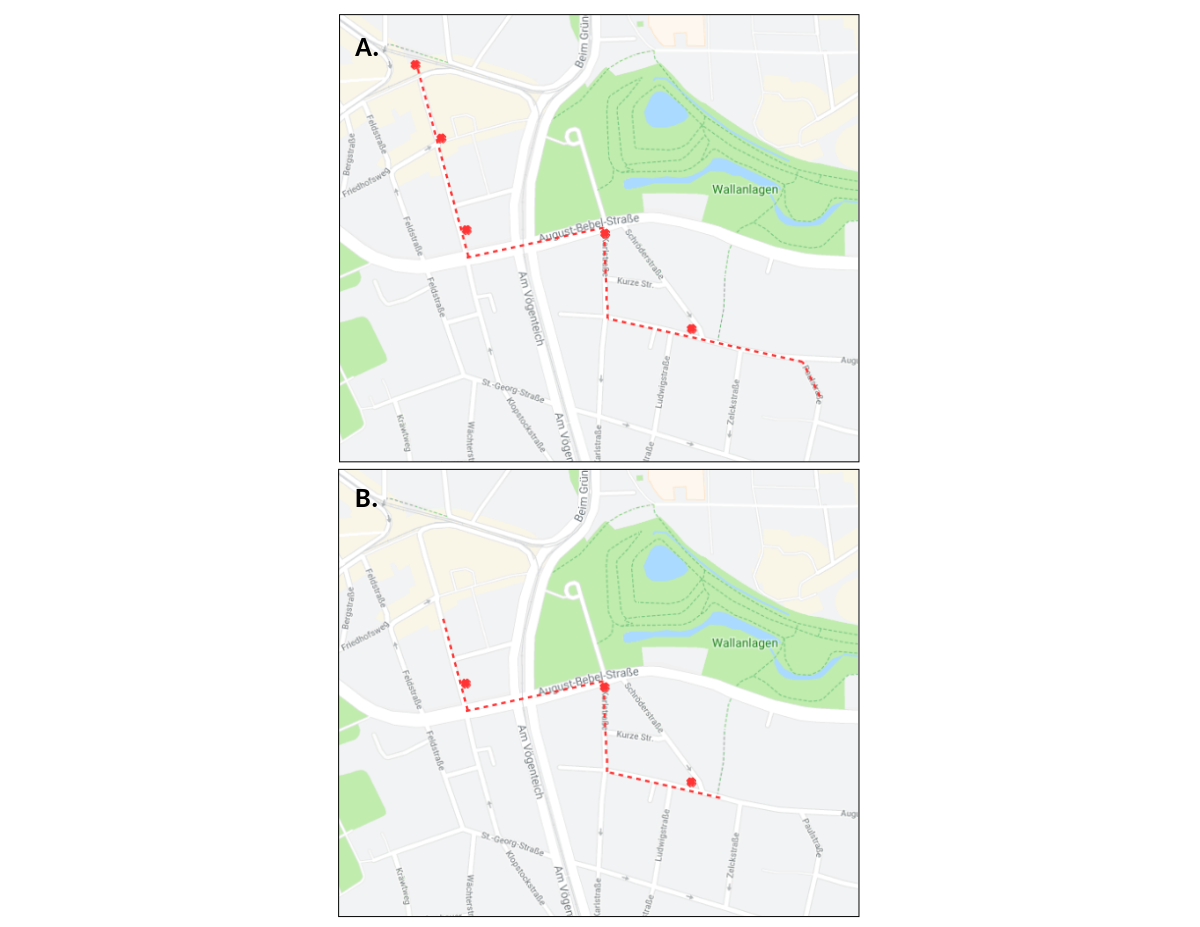
Map of the routes of (A) cognitively healthy older participants and (B) patients with dementia. Red crosses denote locations where the environment is changed in the experimental run. The environment is unchanged for the patients with dementia; however, the route is shortened (image: Google Maps).

**Figure 4:**
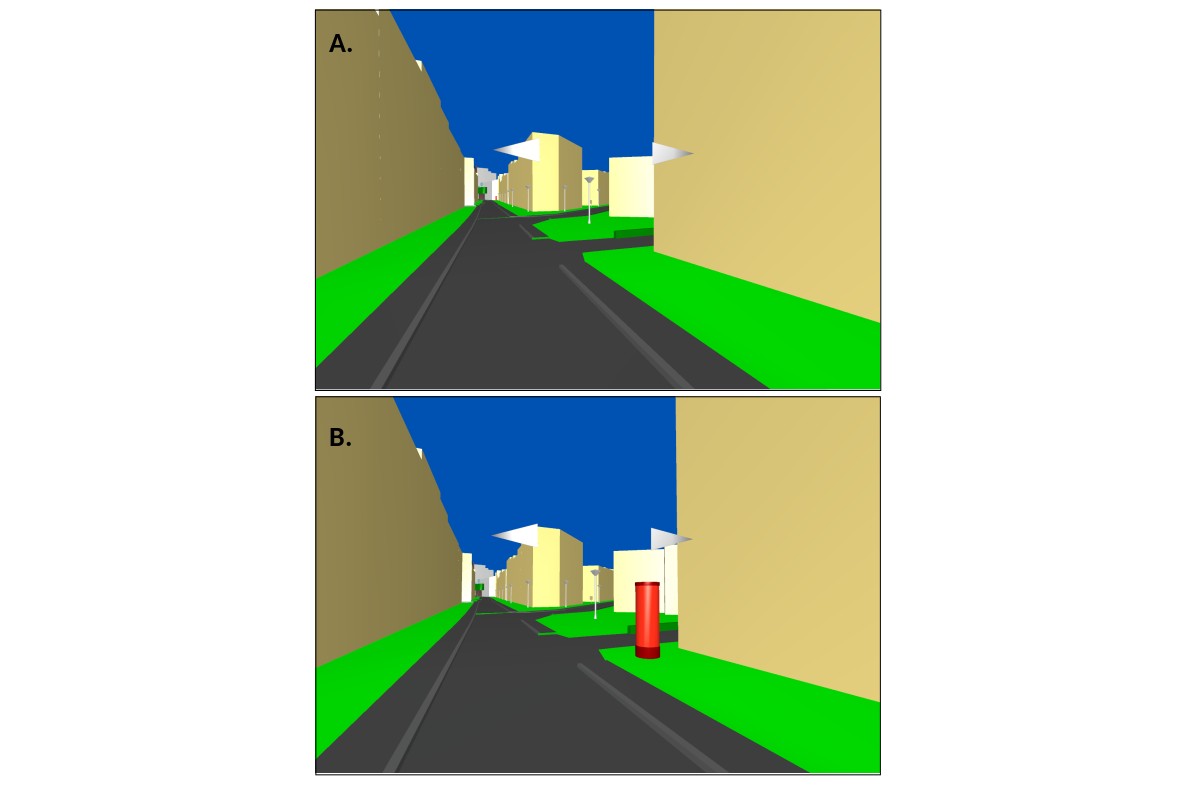
Example of changes in the environment to induce disorientation. (A) Original environment shown in the guided walk (note the red landmark at the far end of the road, to the right). (B) Manipulated environment used in the experimental run; in this case, the landmark is moved to a different intersection.

Participants were guided along a path in the VR environment (Figure 3). Afterward, they were set back to the starting location and asked to walk the same path again, this time unguided. For half of the healthy young or older subjects (the experimental group), phases of disorientation were induced by changing landmarks or decision points in the VR environment (Figure 4) while subjects were required to walk the path on their own. The changes were (1) moving a landmark from one intersection to the next intersection, (2) adding a decision point (ie, an intersection), (3) blocking a road, or (4) moving the goal indicator to a different location. Overall, 5 locations have been manipulated. For the patients with dementia, the environment was not changed, as we assume that these participants would show phases of disorientation already without such changes due to existing cognitive deficits. Based on the experience with the first patient with dementia, we adapted the route for the other patients by shortening it due to earlier observed fatigue while navigating the route (Figure 3).

The participants were familiarized with the depicted city center by briefly showing them a map, such that problems in wayfinding will be due to disorientation instead of exploration in an unknown environment. In addition to recording kinematic and kinetic gait parameters as provided by the GRAIL system, participants are also equipped with 3 wearable sensors on the left wrist, right ankle, and chest, each of which contain a 3-axes accelerometer and 3-axes gyroscope sampled with 64 Hz. Additionally, the chest sensor records an electrocardiogram (ECG; 1024 Hz), and the wrist sensor records electrodermal activity (EDA; 32 Hz). Wearable sensor data and data provided by the GRAIL system are synchronized by an event-based mechanism (ie, participants perform a distinctive movement at the beginning of the recording, which can be easily located in all sensors) and are resampled to 100 Hz. All wearable sensors have been used in previous studies [6,30] and validated [31-33].

After the orientation task, participants filled out the igroup Presence Questionnaire (IPQ) [34], a questionnaire on the functionality and realism of the interaction with the VR environment, as well as questions regarding engagement in the study, hobbies and activities, technical device usage, and demographics. Additionally, participants provided answers to open questions on (1) problems that occurred in controlling the direction, (2) suggestions for improving the controlling mechanism, (3) which properties in the VR they used for orientation, (4) orientation problems they had, and (5) suggestions for enabling better self-orientation in the VR environment. Table 1 displays the experimental procedure in phases.

**Table 1:** Experimental procedure for the cognitively healthy older participants (n=9) and patients with dementia (n=4). The patient group followed the procedure of the control group.

Phase		Experimental group (n=4)	Patients with dementia (n=4) and control group (n=5)
1	Preparation (90 min)	Study information, informed consent, assessment of physical and cognitive status, blood draw, fixing of markers and electrodes, and practice walking on treadmill	
2	Task 1 (20 min)	Learning the route: accompanied walk (route: Figure 3)	
3	Task 2 (20 min)	Autonomous navigation (modified environment)	Autonomous navigation (unchanged environment)
4	Questionnaires (20 min)	Presence, navigation, orientation, experience with technical devices	

### Randomization

Randomization of the healthy younger and older participants into the experimental or control group was carried out using the program Research Randomizer (Social Psychology Network) [35]. In contrast, the patients with dementia are only assigned to the control group (ie, with the adapted route), as we expect a sufficient number of episodes of disorientation in people with MCI or dementia even without interfering with their environment.

### Behavior Annotation

An offline annotation procedure was applied to the video data recorded during the orientation task, for assessing the observable orientation behavior of the participants using the ELAN 5.8 tool (The Language Archive) [36]. As a coding scheme, we used an adequate adaption of the coding scheme provided by Yordanova et al [30]. The same scheme has been used in the SiNDeM field study [6]. This coding scheme was developed both by domain experts and assistive systems designers, based on interviews, video logs, data from a systematic literature review, and concepts from existing ontologies, for the purpose of providing assistance to people with dementia during their outdoor mobility. Hence, it covers aspects of orientation behavior that are beyond the scope of wayfinding in our VR setup (eg, behaviors associated with attention to traffic). For this reason, we adapted the coding scheme to capture exactly those behaviors that are obtainable within our virtual reality setup.

Specifically, to identify instances of disorientation, we annotated when participants show wandering behavior (ie, nongoal-directed walk), communication behavior (ie, asking for help when disoriented), topological orientation (ie, trying to orient themselves based on the surrounding environment), or spatial orientation (ie, trying to orient themselves based on landmarks). In addition, different types of errors that are associated with disoriented behavior were annotated (ie, initiation, realization, sequence, and completion errors). The annotations are being evaluated based on the level of agreement between annotators (ie, interrater reliability in terms of Cohen kappa [37]).

### Ethical Approval

This study has been reviewed and approved by the Ethics Commission of the University Medicine Rostock (Approval number: A 2019-0062).

### Outcome Measures

The study is estimated to run until the end of 2021. The outcome measures to be collected are included in Table 2. However, for this pilot study, we focused on the feasibility and usability (level of immersion, perceived control of the gait system) as primary outcomes, and on measures of motion variations (walking speed), physiological variations (heart rate, skin conductance response), and spatial disorientation (frequency of occurrence) as secondary outcomes.

**Table 2:** Study outcome measures.

Outcome Measure	Measurement	Modality	Status
Feasibility and usability	Level of immersion, usability feedback	igroup Presence Questionnaire (IPQ), usability questionnaire	Ongoing
Heart rate variability	Rate of change in heart rate	Electrocardiographic sensor	Ongoing
Skin conductance	Rate of change in electrodermal response	Electrodermal activity sensor	Ongoing
Spatial disorientation	Incidences of disorientation	Customized annotation scheme in ELAN	Ongoing
Gait variability	Incidences of change in gait pattern	Gait capturing system of the GRAIL	Ongoing
Accelerometry	Incidences of change in motion pattern	Accelerometers	Ongoing
Apolipoprotein E4 status	Presence of the variants Apo-E2, -E3, and -E4 in the blood samples	7.5 ml blood samples	Ongoing

### Data Analysis

Analysis of both the quantitative and qualitative data collected during the course of the study will take place in accordance with predetermined analysis plans; however, for this feasibility study with a limited sample size, we reported basic descriptive statistics (mean and standard deviation as well as frequencies) applied to the quantitative and qualitative data for evaluation purposes using R statistical software (version 3.6.0; R Core Team).

## Results

### Immersiveness of the Virtual Environment

All participants could complete the experiment. Responses to the IPQ informed us that the cognitively healthy older participants perceived an above-average degree of immersion; group mean item scores (between 1 and 7, where 7 means highest perceived presence/involvement/realism) were 4.60 for presence, 3.92 for involvement, and 4.42 for realism. The patients with dementia, on the other hand, while reporting lower mean scores relative to the cognitively healthy older participants, still perceived a considerable degree of immersion, with group mean item scores of 3.63 for presence, 3.31 for involvement, and 2.60 for realism. Of the 13 participants, only 1 (7%) of the participants reported simulator sickness.

### Inducing Disorientation Through the Manipulation of the Virtual Environment

Our setup was viable in inducing instances of disorientation. We observed an average of 21.40 instances of disorientation for the cognitively healthy older participants in the control group and 36.50 instances for the cognitively healthy older participants in the experimental group. For the patients with dementia, an average of 37.50 instances of disorientation was observed. A number of these instances of disorientation were observed either at points where the virtual environment was manipulated or at subsequent points afterward, where the participants had to reorient themselves due to the altered virtual environment (Figure 4). This amounted to a good proportion of the data being annotated as disoriented. Furthermore, regarding properties used for orientation, participants mentioned the landmark shown in Figure 4, houses, intersections, and trees.

### Comfortability With the Walking Pattern Change due to our Navigation Mechanism

Participants’ responses to the usability questionnaire show that the control over the chosen direction in the VR environment was perceived as functional for both the cognitively healthy older participants and patients with dementia (eg, easy to learn; participants were able to move to where they wanted at their own pace), and adequately naturalistic. Table 3 shows the answer scores on the questionnaire items regarding usability and navigation control. When asked about problems that occurred, only 2 (22%) of the 9 cognitively healthy older participants mentioned initial difficulty with controlling the direction of movement, while 1 (25%) of the 4 patients with dementia mentioned that it felt unusual. Of the 9 cognitively healthy older participants, 2 (22%) also mentioned that the right and left movement felt a little rapid. Additionally, in the case of the first patient with dementia sampled, we observed early fatigue while navigating the route, leading to an adaptation of the patient’s route (Figure 3). Also, due to a temporary technical fault at the time of recording, we could not obtain gait information for the first patient with dementia sampled. However, all other data were collected for this patient, and complete data could be collected for the 3 subsequent patients with dementia sampled.

**Table 3:** Questionnaire regarding the usability of controlling movement in the virtual-reality environment for the cognitively healthy older participants (n=9; group mean score) and patients with dementia (n=4; group mean score). The scale ranged from 1 (fully disagree) to 7 (fully agree).

Questionnaire item		Cognitively healthy older participants (n=9), mean score	Patients with dementia (n=4), mean score
1.	Learning to navigate was easy.	4.9	4.0
2.	After a while, I did not have to think about how to navigate.	4.3	3.0
3.	I was able to move where I wanted.	5.8	3.6
4.	I was able to move how I wanted	4.9	3.0
5.	I was able to stop at a specific place when I wanted.	5.9	4.0
6.	I did not feel limited in my freedom of movement.	5.8	3.3
7.	I felt tired after navigating.	3.3	2.5
8.	Navigation felt natural.	4.7	3.3

### Measuring Motion and Physiological Parameters

**Figure 5:**
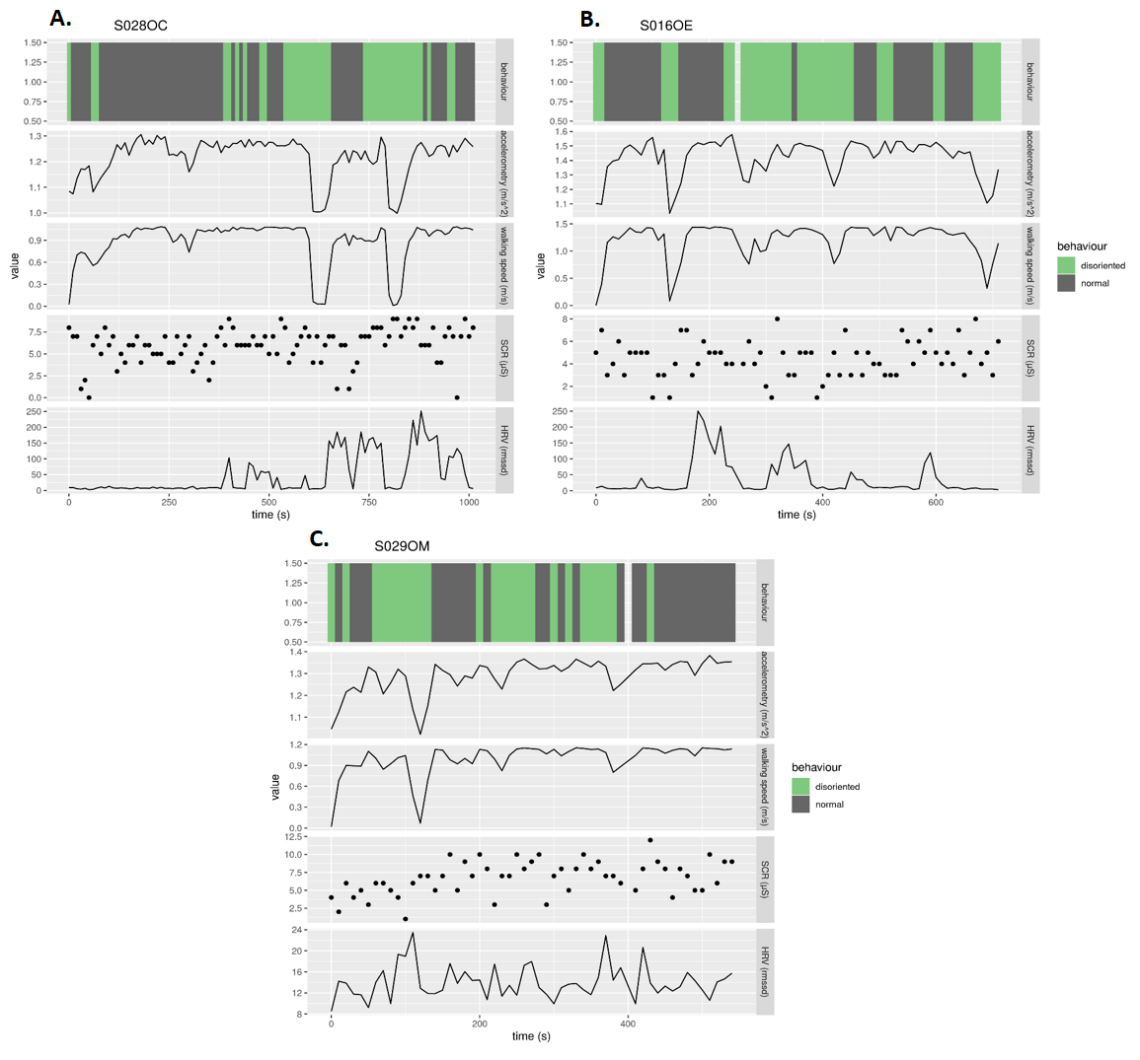
Sample of motion and physiological data showing variations in walking speed, accelerometry, heart rate, and skin conductance of participants occurring during instances of disorientation (green bars in the first row) from the unguided walk for (A) an older control participant (environment not manipulated), (B) an older experimental participant (environment was manipulated), and (C) a patient with dementia (environment not manipulated).

Variations in walking speed, heart rate, and skin conductance response were observed among our pilot participants. Figures 5A, B, and C, respectively, show a sample of the recorded walking speed, accelerometry, heart rate variability, and skin conductance response amplitude for a participant in the control group (where the environment was not manipulated), a participant in the experimental group (where the environment was manipulated, as described above), and for a patient with dementia (where the environment was not manipulated). Relative decrease in motion (walking speed and accelerometry) and an increase in heart rate variability and skin conductance response amplitude can be observed during instances of disorientation.

## Discussion

In this paper, we presented the design for a study investigating the effect of spatial disorientation on physical motion and physiological features, as well as the initial outcomes of the pilot sample. The combined usage of a fully instrumented treadmill, wearable sensors, and an adequately immersive VR system to investigate real-time disorientation among older participants and patients with dementia during active navigation is, to the best of our knowledge, unprecedented. Previous studies that investigated spatial orientation [38-40] and navigation memory [20,41] have relied mostly on VR systems in which navigation or interaction with the VR environment was passively based on the use of control objects (eg, joystick, mouse). This, however, further limits the ecological validity of such setups, as the locomotion aspect of real-world navigation was apparently lacking.

Our patient and cognitively healthy older participant dyad reported a noticeably varying degree of immersion. While the older healthy participant group reported a higher level of immersion [42], the patient group reported a relatively lower level of immersion compared to the cognitively healthy older participant group. However, this is not surprising as we also expect cognitive and perceptual abilities to play a significant role in the evaluation of the VR setup, as people with dementia are known to experience visuoperceptual difficulties [43,44], which may influence this judgment. Nonetheless, participants’ responses to the usability part of the questionnaire indicated an overall confidence that our setup is effective in investigating manifestations of disorientation among cognitively healthy older adults and people with dementia. Furthermore, the first patient with dementia sampled experienced fatigue while navigating the route (akin to the finding of Behrens et al [27]), which suggests that dual-task conditions can be mentally exhausting among older participants. Therefore, this effect could be exacerbated by the increased difficulty of multitasking as a result of cognitive deficits [45]. Hence, we adapted the route of the patients by shortening it relative to the original route.

Considering the number of instances of spatial disorientation, we confirmed our expectation of finding more disorientation events among the participants in the experimental group (changed environment) compared to the participants in the control group (unchanged environment). This preliminary observation gives credence to the validity of our setup in inducing the target behavior of disorientation. We also observed that the patients with dementia expressed a considerable amount of disorientation. This was the case even though the environment remained unchanged, thereby indicating that moments of disorientation accruing to cognitive deficits can also be reliably observed among patients with dementia in our setup. The variations in motion and physiological data observed among our pilot participants illustrates that we are able to adequately obtain the relevant motion and physiological parameters within our approach. As building situation-adaptive devices for supporting navigation among people with dementia relies on the adequate detection of disorientation [6], our setup therefore provides a promising approach to acquiring the needed data.

We acknowledge that there are multiple alternatives to using the GRAIL system for the goal of investigating the effect of spatial disorientation on gait in a lab environment. An alternative to projecting the VR to a screen would be to use a VR headset, like the Oculus Rift (Facebook Inc) or the Samsung Gear VR (Samsung Electronics). A main advantage of the GRAIL over the Oculus Rift and Samsung Gear VR lies in the relative ease of use, safety, and closeness to reality for our study sample, which consists of cognitively healthy older adults and people with dementia who may not be as apt as younger participants at learning to navigate with a VR headset. To participate in the navigation task on the GRAIL, the participant walks freely on the treadmill without any headset or hand gear and observes the virtual environment as one normally would in a real-life situation. In contrast, with the Oculus Rift and Samsung Gear VR, a remarkable difference to the real-world situation is observed the moment one has to put on a headset and navigate with the use of controllers. Additionally, such devices can quickly cause simulator sickness, especially if participants have to move through the environment and when synchronization of head movement and VR movement is not optimal [46]. In this study, simulator sickness was only reported by 1 of the participants so far. Moreover, virtual reality games [such as serious games for dementia care (SGDC)] in which participants interact with the virtual environment using their body movements in the absence of controllers have also reported positive effects on patients’ mental and physical health [47]; these include improvement in memory and motivation, through the performance of mental and physical activities that are carried out while using various physical motions.

A major experience reported by a few of the participants, which also serves as a limitation to the current study, was the fact that control over left/right movement felt somewhat challenging. Possible alternatives to the current direction control mechanism used in this study include using a game controller to choose directions [19], or leaning in the respective direction [48]. However, we expect that this would be perceived as even more unrealistic in terms of how well the experimental setup models the real-world situation in which we are very much interested, as this would require movements that are not representative of real-world locomotion. For instance, Kizony et al [19] acknowledged that the combination of the joystick and treadmill may have posed an additional challenge to the older adults, although no discomfort was reported. This could have been further confirmed if participants were asked about their experience using structured measures such as the IPQ and usability questionnaires as employed in this study. Nonetheless, usability and immersion in VR studies remains an open topic. Further studies replicating the setup employed in this study could serve to provide further testing and data for evaluating this setup. Omnidirectional treadmills [49,50] (that allow free movement in all directions) could be a promising alternative for this purpose. However, such systems are still research prototypes and are not commercially available.

In conclusion, our GRAIL setup allows us to collect a rich dataset: most prominently, the motion capturing system can be used to assess spatio-temporal gait parameters (eg, walking speed). Results from this feasibility study suggest that our setup is sufficient in investigating sensor-derived features of spatial disorientation, even in patients with dementia, in a safe and controlled environment that is comparable to the real world. This is evident in the fact that we observed a considerable amount of disorientation among our participants, which also corresponded to the sensor data and questionnaire responses. Based on the full data set, in the future, we will focus on training machine learning classifier models for discriminating instances of disorientation from moments of orientation, with the aim of coming up with a relevant feature set for developing an interventional device. This classifier subsequently will be tested with real-world data (ie, perform transfer of learning). This feasibility study encourages further investigations on the possibility of more robust real-time detection of spatial disorientation, with the aim of coming up with adequate situation-aware interventions.

## Abbreviations

AD: Alzheimer disease
ATD: assistive technology device
BPM: beats per minute
ECG: electrocardiography
EDA: electrodermal activity
GRAIL: Gait Real-time Analysis Interactive Lab
IPQ: igroup Presence Questionnaire
MCI: mild cognitive impairment
MMSE: Mini–Mental State Examination
OSM: OpenStreetMap
SiNDeM: Situation-Aware Navigation Assistance for Dementia Patients using Causal Behavior Models
VR: virtual reality
